# Reproducibility of the Motor Optimality Score–Revised in infants with an increased risk of adverse neurodevelopmental outcomes

**DOI:** 10.1111/dmcn.16256

**Published:** 2025-02-10

**Authors:** Carly Luke, Arend F. Bos, Michelle Jackman, Robert S. Ware, Anya Gordon, Christine Finn, Dyvonne H. Baptist, Katherine A. Benfer, Margot Bosanquet, Roslyn N. Boyd

**Affiliations:** ^1^ Queensland Cerebral Palsy and Rehabilitation Research Centre, Child Health Research Centre The University of Queensland Brisbane Australia; ^2^ Queensland Paediatric Rehabilitation Service Children's Health Queensland Hospital and Health Service Brisbane Australia; ^3^ Beatrix Children's Hospital, Division of Neonatology University of Groningen Groningen the Netherlands; ^4^ John Hunter Children's Hospital Newcastle Australia; ^5^ Cerebral Palsy Alliance University of Sydney New South Wales Australia; ^6^ Griffith Biostatistics Unit Griffith University Brisbane Australia; ^7^ Department of Physiotherapy Townsville Hospital and Health Service District Townsville Australia; ^8^ Department of Health and Wellbeing Townsville Hospital and Health Service District Townsville Australia

## Abstract

**Aim:**

To determine reproducibility of the Motor Optimality Score–Revised (MOS‐R) to assess infants at high risk of adverse neurodevelopmental outcomes, including cerebral palsy (CP), autism, and developmental delays.

**Method:**

Thirty infants (18 males, 12 females, gestational age mean [range] = 32.5 [23–41] weeks) were randomly selected, according to 2‐year outcome (typically developing; CP; or adverse neurodevelopmental outcome [ad‐NDO]) from a prospective cohort. Participants had two General Movements videos between 12 weeks and 15 + 6 weeks corrected age. Six assessors, masked to history and outcomes, independently scored the MOS‐R from videos. Assessors scored either one (Group 1; *n* = 3) or two videos for each infant (Group 2; *n* = 3). Intraclass correlation coefficient (ICC), Gwet's agreement coefficient, and limits of agreement were calculated.

**Results:**

Combined interassessor reliability (IRR) over six assessors for total MOS‐R was ‘fair’ (ICC = 0.56, 95% confidence interval [CI] 0.41–0.72), and ‘excellent’ with consensus agreement (ICC = 0.99, 95% CI 0.98–0.99). Analyses demonstrated a mean interrater difference of 0.316 (95% limits of agreement −11.51, 12.14) over 450 comparisons (15 pairs). IRR was ‘moderate’ to ‘almost perfect’ across subcategories, with the highest reliability ‘movement patterns’ (Gwet's agreement coefficient = 0.73–1.00) and the lowest ‘postural patterns’ (0.45–0.73). Assessors who scored two videos (Group 2) demonstrated higher reproducibility. IRR for total MOS‐R was ‘excellent’ when infants were typically developing (ICC = 0.90), and ‘good’ for CP (0.74) and ad‐NDO (0.68).

**Interpretation:**

The MOS‐R is a highly reproducible tool for assessing infants at high risk of ad‐NDOs and is feasible for implementation in clinical settings. Reproducibility is best when the tool is used by experienced assessors to gain consensus agreement.

## Graphical abstract

The Motor Optimality Score‐Revised assesses early infant motor repertoire, with promising predictive ability for identifying developmentally vulnerable infants, ensuring good reproducibility is essential for real‐world implementation. We demonstrated high reliability and agreement among six assessors across a cohort of infants with varied neurodevelopmental risk status. Reproducibility was improved with consensus agreement, when clinicians assessed two infant videos consecutively and greatest for infants who were demonstrating a clear trajectory of developmentally ‘on track’.

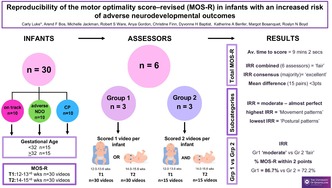

**Abbreviations**
ad‐NDO: adverse neurodevelopmental outcome
GMA: General Movements Assessment
ICC: intraclass correlation coefficient
IRR: interassessor reliability
MOS: Motor Optimality Score
MOS‐R: Motor Optimality Score–Revised
non‐VPT: not born very preterm
VPT: born very preterm

**What this paper adds**

- The Motor Optimality Score–Revised (MOS‐R) is highly reproducible among experienced assessors.
- Gaining a consensus agreement improves reliability for MOS‐R total and subcategory scores.
- Scoring two videos for each infant improves interassessor reliability and agreement.
- Reproducibility is highest when infants are demonstrating a typical developmental trajectory.
- Interassessor agreement for the presence of additional atypical postural patterns is low.

Infants exposed to newborn detectable risk factors have an increased risk of adverse neurodevelopmental outcomes (ad‐NDO).^1^ Adverse outcomes may occur as specific diagnoses, including cerebral palsy (CP),^2^ or as delays across motor, cognitive, communication, and/or behaviour domains,^3^, ^4^, ^5^ with neurodevelopmental vulnerability often not detected until later in infancy. Targeted neonatal surveillance programmes routinely screen high‐risk infants; however, some tools lack the sensitivity to detect subtle differences in movement quality which may identify neurodevelopmental vulnerability before the onset of clinical signs.^6^ Ideally, early screening tools should reliably identify an infant's risk status for a range of ad‐NDOs, including CP, autism, and developmental delays.^7^

Prechtl's General Movements Assessment (GMA) has the strongest predictive validity for the early detection of CP^8^ and has become a vital tool in high‐risk neonatal surveillance clinics.^9^, ^10^ The GMA's predictive accuracy (98% sensitivity, 91% specificity) for CP is well established across research^11^ and real‐world settings;^9^, ^12^ however, its ability to detect other (non‐CP) ad‐NDOs is less sensitive.^6^, ^13^ The Motor Optimality Score (MOS)^14^ is a semi‐quantitative scoring system to objectively assess an infant's movement and postural patterns, in addition to GMA. The MOS involves a more detailed analysis of infant motor repertoire and may differentiate infant risk status for a range of ad‐NDOs.^15^ The revised version (MOS‐R), published in 2019,^16^ has item specific descriptors and updated scoring instructions for the five subcategories: quality of fidgety movements, observed movement patterns, age‐adequate movement repertoire, postural patterns, and movement character. Subcategories are scored separately, then totalled to gain an optimality score. Non‐optimal MOS‐R scores have been associated with later impairments of motor, cognitive, and language in preterm and low birthweight populations, despite the presence of normal fidgety movements.^3^, ^15^ Preliminary evidence supports the MOS‐R as a marker for CP subtype, severity, and topography.^16^

Increased data supporting the predictive ability of the MOS‐R^15^ has enhanced interest in its clinical application, thus good reproducibility is essential for real‐world implementation. Three studies have evaluated interassessor reliability (IRR) of the original MOS,^17^, ^18^, ^19^ demonstrating ‘excellent’ IRR when used to assess infants with genetic conditions^18^, ^19^ and newborn detectable risk factors for ad‐NDOs,^17^, ^18^ with intraclass correlation coefficient (ICC) ranging from 0.86 to 0.96. Fjørtoft et al. additionally reported subcategory agreement as ‘moderate’ to ‘high’.^17^ Recently, two studies^20^, ^21^ have reported IRR between two assessors of the revised version (MOS‐R). IRR was ‘almost perfect’ (ICC = 0.98–0.99) when used to assess infants born in low‐resource communities, extremely preterm, or with prenatal exposure to SARS‐CoV‐2^20^ and ‘excellent’ (ICC = 0.86) in a low‐risk population‐based birth cohort.^21^ Subcategory reliability was ‘substantial’ to ‘almost perfect’ across both studies.

The primary aim of this study was to determine the reproducibility of the MOS‐R total score and subcategories, when assessing infants with a broad range of early risk factors for ad‐NDOs. Based on the high reproducibility of the MOS‐R across extreme preterm birth^22^ and low‐resource populations,^20^ we hypothesized the MOS‐R would be highly reproducible across all assessment ages and risk exposures. Our secondary aim was to explore if reproducibility was impacted by infant factors: gestational age, age at assessment, and developmental outcome at 2 years corrected age and whether reproducibility improved when assessors scored two versus one video for each infant. We hypothesized that reproducibility would be higher among assessors who scored two videos and for infants with a later gestational age, older age at assessment, and clear developmental trajectory (typically developing or high‐risk CP).

## METHOD

### Study design and participants

This assessor masked reproducibility study is nested within a prospective cohort study including 30 participants from the Queensland Early Detection and Intervention Network. The Queensland Early Detection and Intervention Network study recruited infants with an increased risk of CP and/or ad‐NDOs, because of birth (i.e. born preterm [< 37 weeks], low birthweight [< 2500 g], hypoxic‐ischaemic encephalopathy, admission to a neonatal unit), or postneonatal (i.e. head injury, stroke, infection, non‐accidental injury) factors. Informed parental consent was gained (HREC/17/QRCH/83). Infants were screened on the GMA,^8^ with developmental outcomes at 2 years corrected age. Infant outcomes were evaluated from paediatrician diagnosis and/or assessment on the Ages and Stages Questionnaire, Third Edition. Participants were eligible for inclusion in the reproducibility study if they had completed two, scoreable GMA videos at 12 to 13 + 6 weeks and 14 to 15 + 6 weeks, with developmental outcomes at 2 years corrected age (birth years 2017–2020).

### Assessors

Six assessors, masked to infant history and outcomes, independently scored the MOS‐R. Assessor D (AB) was a neonatologist, advanced General Movements Trust trainer, and an expert in GMA and MOS‐R. Assessors A, B, C, E, and F were experienced clinicians who regularly use the MOS‐R. All assessors were advanced GMA trained, with additional MOS‐R training and a minimum 6 months' experience calibrating with an expert (AB) as part of a weekly calibration group.

### Procedure and scoring

Developmental outcomes were classified as typically developing (within 1SD of mean on all Ages and Stages Questionnaire, Third Edition domains and/or no developmental concerns) or confirmed/high risk of (1) CP (paediatrician confirmed/diagnosis of ‘high risk’ CP) or (2) other ad‐NDOs (paediatrician confirmed diagnosis of non‐CP ad‐NDO and/or >1SD below mean on ≥1 Ages and Stages Questionnaire, Third Edition domain), collated by an independent coordinator (CL). GMA video quality was classified as ‘scoreable’ or ‘unscoreable’ based on Kwong et al.^23^ Infants with one or more ‘unscoreable’ video were excluded. Participants were further categorized by gestational age as born very preterm (VPT; < 32 weeks) or not born very preterm (non‐VPT; ≥ 32 weeks). Ten infants were randomly selected from each developmental outcome group (*n* = 5 VPT and *n* = 5 non‐VPT) for inclusion (Figure S1).

Assessors were allocated into two groups: Group 1 (assessors A, B, C) who scored one video for each infant, 15 (50%) at timepoint 1 (T1: 12.0–13 + 6 weeks) and 15 (50%) at timepoint 2 (T2: 14.0–15 + 6 weeks); and Group 2 (D, E, F) who consecutively scored two videos (T1 and T2) for each participant. Assessors independently accessed and scored de‐identified infant videos using the MOS‐R item descriptors, proforma, and method of Einspieler et al.^16^ The five MOS‐R subcategories were scored separately, then totalled to gain an optimality score ranging from 5 to 28 (Figure S1). While the absence of fidgety movements identifies infants with a higher risk of CP,^11^ their presence does not discount an infant's risk of other ad‐NDOs. This subcategory is more heavily weighted, with scores ranging from 1 to 12 points and has the greatest potential to influence differences in total scores. To determine the impact of fidgety movements on reproducibility, MOS‐R total score was analysed with fidgety movements included (range 5–28) and excluded (range 4–16). Total MOS‐R was further classified as optimal or mild, moderate, or severely reduced, to enable consensus agreement, defined as majority agreement of at least 67% (4/6 or 2/3) assessors for MOS‐R classification (Figure S1), to be determined for each participant video. Assessors recorded their time taken to score each video. MOS‐R proformas were transferred to CL who independently collated and entered infant scores into a Research Electronic Data capture (REDCap; Vanderbilt University, Nashville, TN, USA) database. To enable detailed reporting of the association between MOS‐R and 2‐year neurodevelopmental outcomes, all videos were rescored by our entire calibration group a minimum of 2 months after individual assessor scores had been collated (Figure S1). Detailed infant perinatal data was collected from medical charts and recorded in REDcap.

### Statistical analysis

Statistical analyses were completed using Stata, version 17 (StatCorp, College Station, TX, USA). Infant summary statistics are presented as median and interquartile range (IQR) for continuous variables and frequency (%) for categorical variables. Differences between medians for neurodevelopmental outcome groups were determined using quantile regression. For analyses, MOS‐R was defined as summed total score (1) fidgety movements included (range 5–28), (2) fidgety movements excluded (range 4–16), and (3) classified MOS‐R (Figure S1).^16^, ^24^, ^25^ Gwet's agreement coefficient and percentage agreement (%) assessed IRR and agreement respectively for MOS‐R subcategories and classified MOS‐R. Values were interpreted as Gwet's coefficient 0.00 to 0.21 ‘poor’, 0.21 to 0.40 ‘fair’, 0.41 to 0.60 ‘moderate’, 0.61 to 0.80 ‘substantial’, and 0.81 to 1.00 ‘almost perfect’.^26^ ICC two‐way mixed effects model (1, 3) determined IRR of total MOS‐R with ICC interpreted as 0.75 to 1.00 ‘excellent’, 0.60 to 0.74 ‘good’, 0.40 to 0.59 ‘fair’, and less than 0.40 ‘poor’ reliability.^27^ Bland–Altman limits of agreement were used to determine the interrater agreement reported as the mean difference in average MOS‐R score between the two assessors (15 pairs over 450 pairwise comparisons), based on the assumption that observations were independently distributed. We will present limits of agreement, which is the range that 95% of results are expected to lie between.

Reproducibility (IRR and agreement) was evaluated for combined (six assessors), pairwise comparisons (15 pairs), consensus (assessor scores whose MOS‐R was classified within majority agreement), Group 1 (1 video) versus Group 2 (2 videos), age at time of assessment (T1 vs T2), gestational age (VPT vs non‐VPT), and neurodevelopmental outcome (typically developing vs. ad‐NDO vs CP) (Figure S1). For comparisons between Group 1 and Group 2, analyses included scores from the 30 infant videos scored by all six assessors.

## RESULTS

Total 58 of 104 (56%) Queensland Early Detection and Intervention Network participants met inclusion for the reproducibility study (Figure S1) and were classified as typically developing (*n* = 15, 26%) or high risk/confirmed ad‐NDO (*n* = 22, 38%) or CP (*n* = 21, 36%). Eight (14%) infants were excluded because of poor video quality (Table S1). Characteristics for the 30 included infants (18 males, 12 females, gestational age mean [SD] =32.5 [4.5] weeks) are summarized in Table 1. At T1 (12.0–13 + 6 weeks) infants were classified on MOS‐R as optimal (*n* = 3, 10%), non‐optimal (*n* = 18, 60%), moderately reduced (*n* = 5, 17%), and severely reduced (*n* = 4, 13%). At T2 (14.0–15 + 6 weeks) infants were classified as optimal (*n* = 2, 7%), non‐optimal (*n* = 19, 63%), moderately reduced (*n* = 2, 7%), and severely reduced (*n* = 7, 23%) (Table S2).

**TABLE 1:** Participant risk factors and demographics (*n* = 30 infants).

	*n* = 30
Gestational age (weeks.days), mean (SD)	32.5 (5.6)
VPT (*n* = 15), *n* (%)	27.4 (2.3)
non‐VPT (*n* = 1 5), *n* (%)	37.5 (2.4)
Birthweight (g), mean (SD)	2048.8 (1167.1)
Resuscitation at birth, *n* (%)	
None	11 (36.7)
Minor	12 (40.0)
Major	7 (23.3)
Hypoxic‐ischaemic encephalopathy, *n* (%)	4 (13.3)
Therapeutic cooling	3 (10.0)
Seizures, *n* (%)	8 (26.7)
Bronchopulmonary dysplasia, *n* (%)	8 (26.7)
Multiple birth, *n* (%)	2 (6.7)
Surgery, *n* (%)	4 (13.3)
Cardiac	1 (3.3)
Gastroschisis	0 (0.00)
Ventriculoperitoneal shunt	1 (3.3)
Other	2 (6.7)
Postneonatal injury, *n* (%)	
Infection	6 (20.0)
Head injury	0 (0.0)
Non‐accidental injury	0 (0.0)
Syndrome, *n* (%)	2 (6.7)
Sex, *n* (%)	
Male	18 (60.0)
Female	12 (40.0)
Neuroimaging, *n* (%)	
Cranial ultrasound	21 (70.0)
Magnetic resonance imaging	15 (50.0)
Intraventricular haemorrhage, *n* (%)	6 (20.0)
Periventricular leukomalacia, *n* (%)	2 (6.7)
Stroke, *n* (%)	2 (6.7)
General Movements Assessment age, mean (SD)	
T1 (weeks.days)	12.3 (0.4)
T2 (weeks.days)	14.4 (0.5)

Abbreviations: non‐VPT = not very preterm (>32 weeks); T1, timepoint 1 (12–13 + 6 weeks); T2, timepoint 2 (14–15 + 6 weeks); VPT, very preterm (<32 weeks).

Assessors took on average 9 minutes 2 seconds (SD = 3.09 minutes) to score the MOS‐R, with the expert assessor (assessor D) the fastest (mean = 6.07 minutes, SD = 1.90 minutes).

### Reproducibility

**FIGURE 1:**
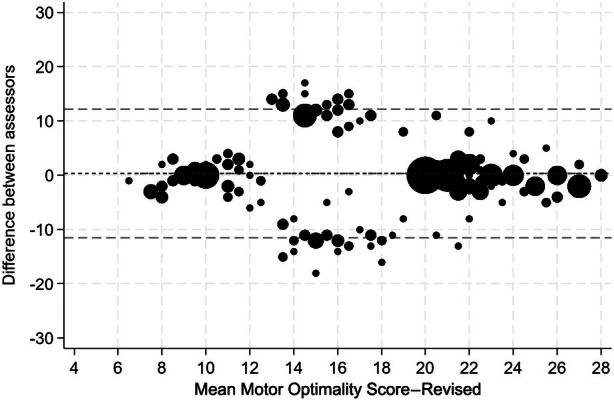
Bland–Altman limits of agreement plot of the agreement between assessors (6 assessors, 15 pairs) for the total Motor Optimality Score–Revised (*n* = 450 pairwise comparisons).

For total MOS‐R, combined interrater agreement (six assessors) was good, with pairwise analyses (15 pairs, 450 pairwise comparisons) demonstrating a mean interrater difference of 0.32 (95% limits of agreement −11.51, 12.14, Figure 1). The IRR for total MOS‐R is reported in Table 2. Combined IRR was ‘fair’ (ICC = 0.56, 95% CI 0.41–0.72), for ‘fidgety movements included’ with pairwise comparisons ranging from ‘poor’ to ‘good’ (0.38–0.70). For MOS‐R ‘fidgety movements excluded’, combined IRR improved to ‘good’ (0.69, 95% CI 0.56–0.81) with pairwise comparisons ‘fair’ to ‘excellent’ (0.57–0.79). IRR was ‘good’ (ICC = 0.64, 95% CI 0.44–0.79) for Group 2 assessors (scored two videos) compared to ‘fair’ (ICC = 0.57, 95% CI 0.36–0.74) for Group 1 (scored one video). For MOS‐R subcategories (Table 3), combined IRR (six assessors) was ‘moderate’ to ‘almost perfect’ (Gwet's coefficient = 0.60–0.89) with ‘observed movement patterns’ (Gwet's coefficient = 0.89, 95% CI 0.81–0.97) and ‘movement character’ (Gwet's coefficient = 0.88, 95% CI 0.79–0.97) demonstrating the highest reliability and postural patterns (Gwet's coefficient = 0.60, 95% CI 0.44–0.76) the lowest.

**TABLE 2:** Interassessor reliability of the total Motor Optimality Score‐Revised (MOS‐R), fidgety movements included and excluded, reporting ICC and 95% CIs (*n* = 30 videos).

Assessors	Fidgety movements included		Fidgety movements excluded	
	ICC (1, 3)	95% CI	ICC (1, 3)	95% CI
A, B	0.68	0.42–0.83	0.72	0.49–0.86
A, C	0.70	0.47–0.85	0.67	0.42–0.83
A, D	0.59	0.30–0.78	0.70	0.45–0.84
A, E	0.41	0.06–0.67	0.64	0.37–0.81
A, F	0.52	0.21–0.74	0.57	0.28–0.77
B, C	0.59	0.30–0.78	0.72	0.50–0.86
B, D	0.59	0.30–0.78	0.69	0.45–0.84
B, E	0.51	0.19–0.73	0.79	0.61–0.90
B, F	0.64	0.37–0.81	0.67	0.41–0.83
C, D	0.60	0.32–0.79	0.79	0.60–0.90
C, E	0.48	0.15–0.71	0.78	0.60–0.89
C, F	0.38	0.03–0.65	0.66	0.40–0.82
D, E	0.69	0.44–0.84	0.67	0.41–0.83
D, F	0.58	0.28–0.77	0.74	0.52–0.87
E, F	0.47	0.14–0.71	0.71	0.47–0.85
A, B, C, D, E, F	0.56	0.41–0.72	0.69	0.56–0.81
Gr1 (A, B, C)	0.57	0.36–0.74	0.70	0.53–0.83
Gr2 (D, E, F)	0.64	0.44–0.79	0.68	0.50–0.82
Consensus^a^	0.99	0.98–0.99	0.89	0.81–0.94
T1 (12.0–13.6w)^b^	0.55	0.34–0.78	0.52	0.31–0.76
T2 (14.0–15.6w)^b^	0.52	0.31–0.76	0.82	0.68–0.93
VPT^b^	0.52	0.30–0.75	0.69	0.51–0.86
Non‐VPT^b^	0.61	0.41–0.82	0.71	0.53–0.87

Abbreviations: CI, confidence interval, Gr1, assessors scored one video; Gr2, assessors scored two videos; ICC, intraclass correlation coefficient; non‐VPT, not born very preterm (> 32 weeks); T1, timepoint 1; T2, timepoint 2; VPT, born very preterm (< 32 weeks).

^a^ Consensus = assessor scores whose MOS‐R was classified within majority agreement.

^b^ *n* = 15 videos scored.

**TABLE 3:** Interassessor reliability for MOS‐R subcategories, reporting Gwet's agreement coefficient and 95% CI (*n* = 30 infants).

	MOS‐R subcategories					
Assessors	Fidgety movements GC (95% CI)	Movement patterns GC (95% CI)	Age‐adequate repertoire GC (95% CI)	Postural patterns GC (95% CI)	Movement character GC (95% CI)	MOS‐R classification GC (95% CI)
A, B	0.84 (0.67–1.00)	0.93 (0.82–1.00)	0.47 (0.21–0.74)	0.58 (0.33–0.84)	0.93 (0.82–1.00)	0.76 (0.57–0.95)
A, C	0.83 (0.66–1.00)	0.93 (0.82–1.00)	0.62 (0.37–0.86)	0.66 (0.43–0.89)	0.93 (0.83–1.00)	0.69 (0.49–0.89)
A, D	0.65 (0.41–0.88)	0.74 (0.54–0.94)	0.56 (0.31–0.82)	0.55 (0.27–0.82)	0.82 (0.65–0.98)	0.49 (0.24–0.74)
A, E	0.56 (0.31–0.81)	0.81 (0.65–0.98)	0.62 (0.37–0.87)	0.50 (0.22–0.78)	0.80 (0.61–1.00)	0.58 (0.35–0.81)
A, F	0.63 (0.39–0.87)	0.82 (0.65–0.98)	0.61 (0.37–0.87)	0.63 (0.39–0.87)	0.89 (0.75–1.00)	0.60 (0.38–0.82)
B, C	0.74 (0.54–0.95)	1.00	0.62 (0.39–0.86)	0.62 (0.38–0.86)	0.93 (0.83–1.00)	0.72 (0.52–0.92)
B, D	0.65 (0.42–0.88)	0.93 (0.82–1.00)	0.61 (0.37–0.86)	0.45 (0.17–0.73)	0.85 (0.70–1.00)	0.53 (0.30–0.76)
B, E	0.56 (0.31–0.81)	1.00	0.72 (0.50–0.93)	0.68 (0.46–0.91)	0.92 (0.80–1.00)	0.55 (0.31–0.78)
B, F	0.68 (0.46–0.91)	1.00	0.86 (0.69–1.00)	0.58 (0.34–0.83)	0.85 (0.68–1.00)	0.63 (0.41–0.85)
C, D	0.75 (0.56–0.95)	0.86 (0.70–1.00)	0.66 (0.42–0.90)	0.73 (0.51–0.94)	0.85 (0.70–1.00)	0.69 (0.49–0.89)
C, E	0.67 (0.45–0.89)	0.89 (0.77–1.00)	0.52 (0.25–0.78)	0.68 (0.45–0.91)	0.84 (0.66–1.00)	0.57 (0.35–0.79)
C, F	0.57 (0.32–0.82)	0.89 (0.77–1.00)	0.52 (0.25–0.78)	0.71 (0.50–0.92)	0.85 (0.68–1.00)	0.45 (0.22–0.69)
D, E	0.68 (0.44–0.91)	0.82 (0.65–0.98)	0.76 (0.55–0.97)	0.55 (0.28–0.82)	0.87 (0.72–1.00)	0.60 (0.38–0.82)
D, F	0.60 (0.36–0.84)	0.73 (0.53–0.94)	0.51 (0.25–0.78)	0.51 (0.24–0.78)	0.87 (0.72–1.00)	0.44 (0.21–0.67)
E, F	0.56 (0.31–0.81)	0.89 (0.77–1.00)	0.56 (0.31–0.82)	0.59 (0.35–0.84)	0.91 (0.78–1.00)	0.52 (0.30–0.75)
A, B, C, D, E, F	0.67 (0.53–0.80)	0.89 (0.81–0.97)	0.61 (0.46–0.77)	0.60 (0.44–0.76)	0.88 (0.79–0.97)	0.60 (0.47–0.72)
Gr 1 (A, B, C)	0.61 (0.41–0.81)	0.82 (0.68–0.96)	0.61 (0.43–0.80)	0.55 (0.35–0.75)	0.87 (0.77–1.00)	0.52 (0.35–0.69)
Gr 2 (D, E, F)	0.80 (0.66–0.95)	0.95 (0.88–1.00)	0.57 (0.36–0.78)	0.62 (0.42–0.82)	0.93 (0.85–1.00)	0.73 (0.58–0.88)
Consensus^a^	1.00	0.93 (0.85–1.00)	0.84 (0.70–0.98)	0.77 (0.62–0.93)	0.95 (0.88–1.00)	1.00
T1 (12.0–13.6w)	0.62 (0.46–77.2)	0.84 (0.70–0.99)	0.58 (0.33–0.80)	0.59 (0.35–0.84)	0.95 (0.88–1.00)	0.63 (0.50–0.76)
T2 (14.0–15.6w)	0.72 (0.49–0.95)	0.93 (0.85–1.00)	0.83 (0.67–0.99)	0.61 (0.38–0.84)	0.80 (0.62–0.99)	0.64 (0.45–0.86)
VPT	0.62 (0.41–0.83)	0.84 (0.71–0.97)	0.58 (0.34–0.82)	0.66 (0.43–0.89)	0.85 (0.70–0.99)	0.50 (0.31–0.69)
Non‐VPT	0.71 (0.52–0.90)	0.94 (0.83–1.00)	0.65 (0.44–0.86)	0.54 (0.30–0.78)	0.91 (0.76–1.00)	0.69 (0.53–0.86)

Abbreviations: CI, confidence interval; GC, Gwet's agreement coefficient; Gr1, assessors scored one video; Gr2, assessors scored two videos; MOS‐R, Motor Optimality Score–Revised; non‐VPT, not born very preterm (>32 weeks); T1, timepoint 1; T2, timepoint 2; VPT, born very preterm (< 32 weeks).

^a^ Consensus = assessor scores whose MOS‐R was classified within majority agreement.

Consensus IRR (assessor scores whose MOS‐R were classified within majority agreement) was ‘excellent’ for both total MOS‐R (ICC = 0.99, 95% CI 0.98–0.99) and subcategories (0.77–1.00). Subcategory reliability varied for pairwise, Group 1 versus Group 2, assessment age (T1 vs T2), and gestational age analyses (Table 3), with IRR consistently higher for ‘observed movement patterns’ (Gwet's coefficient = 0.73–1.00) and lower for ‘observed postural patterns’ (Gwet's coefficient = 0.45–0.73) across all comparisons. Agreement for the presence of additional atypical postures was ‘poor’ to ‘fair’ across all analyses (Table S3). Group 2 demonstrated a higher consistency of scoring, with 85.61% of MOS‐R scores differing by 2 points or less between assessors, comparatively, 74.43% of Group 1 scores differed by 2 points or less between assessors (Table S4).

### Reproducibility of the MOS‐R according to neurodevelopmental outcomes at 2 years

Combined IRR for total MOS‐R (Table S5) was ‘excellent’ when infants were typically developing (ICC = 0.90, 95% CI 0.77–0.97) and ‘good’ for infants with confirmed/high risk of CP (ICC = 0.74, 95% CI 0.37–0.93) or ad‐NDOs (ICC = 0.68, 95% CI 0.24–0.91). Detailed infant MOS‐R total and subcategory scores (Table S2) show higher median MOS‐R scores for typically developing infants at both assessment timepoints compared to those at high risk of CP and ad‐NDO, who demonstrate median optimality scores below the clinically significant cut‐off. Videos of typically developing infants were quicker to score on average (Table S4).

## DISCUSSION

The MOS‐R is an observer‐based, semi‐quantitative assessment, which relies on individual Gestalt perception. The revised MOS now includes item descriptors for scoring atypical and typical movement and postural patterns, and an objective means of scoring age‐adequate movement repertoire, which aims to improve IRR among trained assessors. This study found the combined reliability over six assessors for total MOS‐R was ‘fair’ and improved to ‘excellent’ with consensus agreement. Assessors who scored two videos per infant demonstrated greater IRR and agreement than those who only scored one video per infant, indicating that scoring MOS‐R over a trajectory is superior. Subcategory IRR was ‘moderate’ to ‘almost perfect’, with ‘observed movement patterns’ and ‘movement character’ the highest reliability and ‘postural patterns’ the lowest. The IRR for total MOS‐R was ‘excellent’ when infants were typically developing and ‘good’ for those with high risk/confirmed diagnosis of CP or ad‐NDO.

We demonstrated individual variance within total MOS‐R, subcategory, and item‐specific scoring, supporting the need for continued practice and calibration once trained.^18^ Overall IRR for total MOS‐R (six assessors) was fair (ICC <0.60), which was lower in comparison to other reported studies (ICC >0.86).^20^, ^21^, ^22^ Our lower reliability may be explained by a much higher number of assessors per video (6 vs 2), therefore more vulnerable to scoring variability and the impact of outliers. In addition, our study included high‐risk infants with a range of neurodevelopmental outcomes and variable MOS‐R presentations, in comparison to Alexander et al.^21^ who reported reproducibility in a low‐risk population, where only 2 of 773 infants scored absent fidgety. Another important consideration relates to assessor training. In our study, assessors were trained by multiple different tutors, which resembles real‐world clinical experiences; however, this is a key difference to Örtqvist et al.'s study^20^ where assessors were trained by the same tutor and thoroughly discussed the MOS‐R item‐specific definitions before scoring.

To provide recommendations for real‐world implementation, we wanted to replicate current clinical practice for GMA scoring. In many tertiary centres, there are multiple GMA trained clinicians, ensuring results are not based on individual Gestalt perception, rather a consensus agreement (i.e. majority agreement on GMA quality). In our study consensus was defined as a majority agreement of at least 67% (4/6 or 2/3) assessors for MOS‐R classification. Reproducibility improved markedly when analysis included only the MOS‐R from assessors who scored within consensus agreement for each video. Consensus IRR was ‘excellent’ for total MOS‐R and ‘substantial’ to ‘almost perfect’ across subcategories. These results are similar to those reported previously for total MOS‐R^15^, ^20^, ^21^, ^22^ and subcategory agreement^20^, ^21^ demonstrating the importance of scoring regularly within a group and/or having multiple assessors to reach a consensus agreement. Our subcategory results are higher overall than those reported on the original MOS study,^17^ supporting the improved objectiveness of the revised version.

### Postural patterns

Similar to previous studies,^17^, ^20^ the subcategory ‘observed postural patterns’ demonstrated the lowest reproducibility, with little difference seen across timepoints (T1 vs T2), and for number of videos scored per infant. Örtqvist et al.^20^ hypothesized this may be due to the decreased number of subcategory items, where disagreement in one pattern can greatly impact the score. Our assessors demonstrated almost perfect agreement (83.5–94.4%) across the four compulsory postural patterns (head‐centred, body symmetry, presence of asymmetric tonic neck reflex, and variability of finger postures), suggesting that score variation may be due to discrepancies in the additional atypical patterns.

Interassessor agreement for ‘postural patterns’ was slightly higher for infants with CP and ad‐NDO compared to typically developing infants, especially at T2. This difference may be explained by the lower subcategory scores demonstrated by vulnerable infants; 85% of infants at high risk of CP or ad‐NDO scored ‘1’ (atypical > typical postures) compared to typically developing infants who demonstrated greater score variability at both timepoints. At T2, 60% of typically developing infants scored optimally for postural patterns and variability of finger postures, compared to infants with CP (< 10%) or ad‐NDO (< 20%). These findings are consistent with previous studies where a lower postural patterns score may be indicative of later ad‐NDO and/or CP with decreased variability in finger postures a potential marker for later cognitive difficulties.^15^

### Exclusion of subcategory ‘fidgety movements’

MOS‐R reliability increased when ‘fidgety movements’ were excluded from the total score. The MOS‐R demonstrates utility in detecting infants with later motor and cognitive delay,^3^, ^15^ even when fidgety movements are excluded^22^ and could be critical in assisting clinician decisions for referral to early intervention when there is a lack of agreement in the presence of fidgety movements. Our group demonstrated an improvement in IRR across all subanalyses when fidgety movements were excluded. At T2, IRR dramatically improved from ‘fair’ to ‘excellent’, demonstrating the influence of fidgety movements on total MOS‐R where total scores may differ by up to 11 points based on fidgety movements alone.

### Number of videos scored (one vs two)

To our knowledge no study has evaluated the effect of infant factors, gestational age, and developmental outcome on the reproducibility of MOS‐R or whether a difference in agreement exists between assessors who score one versus two videos for each infant. Assessors who scored two infant videos (Group 2) demonstrated higher reliability for total MOS‐R and, with the exception of ‘age‐adequate movement repertoire’, higher subcategory agreement. Notable between group differences were ‘fidgety movements’ (almost perfect vs substantial), ‘postural patterns’ (substantial vs moderate), and MOS‐R classification (substantial vs moderate). While previous studies^20^, ^22^ and our consensus scores demonstrate strong reproducibility regardless of the number of videos scored per infant, this finding has implications for the real‐world implementation of the MOS‐R, where assessing two videos may improve clinician confidence and their understanding of an infant's developmental trajectory over time. Some assessors commented they would be wary of making a clinical decision based solely on one infant video or without a group consensus. In addition to improving clinician confidence, Group 2 scored MOS‐R on average 1 minute 50 seconds faster than Group 1, which is important when considering clinician workload.

### Gestational age

Reliability was lower for infants born VPT (≤ 32 weeks) with IRR for total MOS‐R ‘fair’ compared to ‘good’ for infants born non‐VPT. Similarly, reliability across subcategories was slightly reduced for infants born VPT, with only ‘postural patterns’ demonstrating higher agreement. Studies have reported significantly lower MOS‐R for infants born preterm compared to their term‐born peers.^24^, ^28^ This may reflect non‐optimal brain organization because of increased risk of ad‐NDO, or delayed neuromaturation, consistent with preterm trajectory findings on other neurological assessments.^29^ Our cohort of infants born non‐VPT had a mean gestational age of 37 weeks 5 days (SD = 2.3 weeks) and may have demonstrated a clearer developmental trajectory (either typical or high risk) compared to their VPT‐born peers.

### Neurodevelopmental outcomes at 2 years

Typically developing infants demonstrated median MOS‐R scores above the clinically significant cut‐off (< 21) used to indicate referral to intervention services^15^ and the strongest combined IRR, followed by infants with high risk of CP who had the lowest median scores. This suggests that reliability is higher when differences in motor repertoire and quality of movement are clearly typical (higher scores) or more concerning (lower scores). Comparatively, IRR was lowest for infants with ad‐NDO (middle range score), whose developmental trajectories are less clear and more variable, demonstrated in Figure 1. Similar findings were reported on the original MOS, where IRR among four assessors was impacted by scoring inconsistencies for infants who scored in the middle of the MOS scale.^17^ This reflects our group's anecdotal clinical experience, where prolonged discussion or disagreement regarding the temporal organization and quality of fidgety movements often flags infants who require closer monitoring, even if consensus is for fidgety present. Our findings are limited by a small sample size; however, it is positive to note that no infants with confirmed/high risk of CP or ad‐NDO were classified as optimal on MOS‐R at any timepoint (i.e. no false positives).

### Age at time of assessment

Reproducibility was similar across both assessment timepoints, suggesting infant age at assessment (T1 vs T2) does not impact MOS‐R reproducibility. Subcategory IRR ranged from ‘substantial’ to ‘almost perfect’, for both timepoints, with only ‘age‐adequate movement repertoire’ demonstrating significantly higher IRR at T2 compared to T1 (almost perfect vs moderate). This supports findings by Örtqvist et al.,^20^ who reported high reliability across all assessment age groups (9–25 weeks) and is encouraging when considering clinical implementation where multiple factors may influence the timing of GMA assessment. The differences in ‘age‐adequate movement repertoire’ are likely due to the more objective scoring method at T2, with scores influenced by the presence of two specific movement patterns, foot‐to‐foot and hand‐to‐hand contact, in comparison to T1 where scoring is more reliant on the clinician's ability to identify four normal movement patterns. The presence and quality of foot‐to‐foot and hand‐to‐hand were clearer at T2. No typically developing infants demonstrated atypical foot‐to‐foot contact at T2 compared to two at T1. Interestingly, only typically developing infants  demonstrated typical hand‐to‐hand contact at T1, suggesting that an earlier presence of upper limb midline movements may be a key indicator of a typically developing trajectory.

### Strengths and limitations

Our sample size was adequate to determine the reproducibility of the MOS‐R, with 180 assessments completed across six assessors and an additional 90 completed across three assessors. With only 30 infants included, we were unable to analyse the predictive relationship between MOS‐R and 2‐year developmental outcomes. Our assessors were advanced GMA trained clinicians and researchers, highly familiar with the MOS‐R, with the majority calibrating together for 2 years, which should be considered in the generalizability of our findings. Our findings support the importance of gaining a consensus agreement; however, this may not be feasible for clinicians who are the only trained assessor at their site. This highlights the need to build a community of practice, providing additional assessors for sites with limited access to advanced‐trained clinicians.

### Conclusion

The results of our study support the MOS‐R as a highly reproducible and feasible tool that enhances clinician understanding of infant motor repertoire during the fidgety period, regardless of infant age at assessment. Similar to other studies, we demonstrated good reproducibility across a cohort of infants with varied neurodevelopmental risk status, which is encouraging for real‐world implementation. Reproducibility was improved when clinicians assessed two infant videos consecutively and greatest for infants who were demonstrating a clear trajectory of typical development. We recommend assessing at least two videos taken between 12‐weeks and 16‐weeks corrected age, scored consecutively to improve clinician understanding of the infant's overall neuromotor profile and trajectory. Assessments should be completed independently by clinicians masked to infant risk status, with group consensus agreement used for clinical decisions. Regular calibration is essential to maintain skills, especially for identifying less common postural and movement patterns.

## Supporting information


**Figure S1:** Flow of study and statistical analyses.

**Table S1:** Assessment of GMA video quality, excluded cases.

**Table S2:** Total MOS‐R and subcategory scores by neurodevelopmental outcome at 2 years.

**Table S3:** Interassessor agreement for MOS‐R subcategory ‘observed postural patterns’, agreement, and 95% CIs.

**Table S4:** Group 1 vs Group 2 MOS‐R reproducibility and time taken to score.

**Table S5:** Interassessor reliability for MOS‐R total and subcategory agreement by outcome at 2 years.

## Data Availability

The data that support the findings of this study are available from the corresponding author upon reasonable request.
