# Supplementary material for: Reproducibility of the Motor Optimality Score–Revised in infants with an increased risk of adverse neurodevelopmental outcomes
Source: Dev Med Child Neurol. 2025 Feb 10;67(9):1176–85. doi: 10.1111/dmcn.16256 (PMC12336395; doi:10.1111/dmcn.16256)
Supplement: Supplementary file 1 — Figure S1: Flow of study and statistical analyses. [file DMCN-67-1176-s005.pdf]

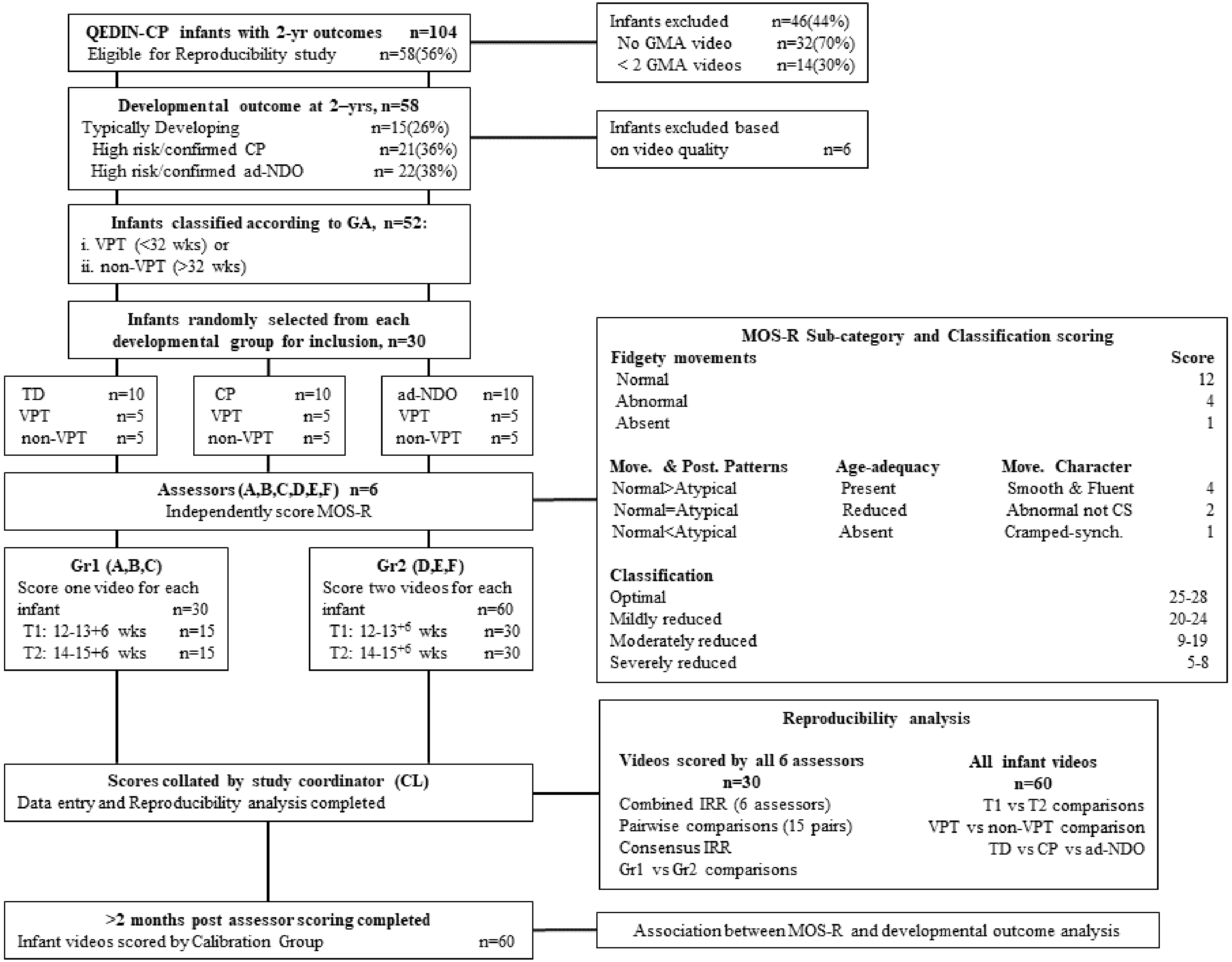

Figure S1: Flow of study and statistical analyses

Abbreviations: ad-NDO=adverse neurodevelopmental outcome, CP=Cerebral Palsy, CS=cramped synchronised, GMA=General movements assessment, Gr1=group 1, Gr2=group 2, IRR=inter-assessor reliability, move.=movement, MOS-R= Motor Optimality Score-revised, TD=typical developing, post.=postural, QEDIN-CP= QLD early detection and intervention network, T1=video 1(12-13.6 wks), T2=video 2 (14-15.6 wks), VPT= very preterm, wks=weeks, yr=year
