# Supplementary material for: Reproducibility of the Motor Optimality Score–Revised in infants with an increased risk of adverse neurodevelopmental outcomes
Source: Dev Med Child Neurol. 2025 Feb 10;67(9):1176–85. doi: 10.1111/dmcn.16256 (PMC12336395; doi:10.1111/dmcn.16256)
Supplement: Supplementary file 2 — Table S1: Assessment of GMA video quality, excluded cases. [file DMCN-67-1176-s002.docx]

Table S1: Assessment of GMA video quality, excluded cases, n=6 infants, 12 videos

| Case | Not positioned in supine | Crying/fussing | External stimulation | Whole body/ limbs not in view | Poor video quality | Short video (<3 minutes) | Outcome at 2 years CA |
| --- | --- | --- | --- | --- | --- | --- | --- |
| Case 1 |  |  |  |  |  |  | Adverse NDO |
| V1  V2 | ^  ^ | ^  ^ | ^  *dummy in mouth | ^  ^ | ^  ^ | ^  ^ |  |
| Case 2 |  |  |  |  |  |  | Adverse NDO |
| V1  V2 | ^  ^ | ^  ^ | *visually engaged  ^ | ^  ^ | ^  ^ | ^  ^ |  |
| Case 3 |  |  |  |  |  |  | CP |
| V1  V2 | ^  ^ | ^  ^ | ^  ^ | ^  ^ | *blurry video  ^ | ^  ^ |  |
| Case 4 |  |  |  |  |  |  | TD |
| V1  V2 | *  ^ | *crying  ^ | ^  ^ | ^  ^ | ^  ^ | ^  ^ |  |
| Case 5 |  |  |  |  |  |  | Adverse NDO |
| V1  V2 | ^  ^ | ^  ^ | ^  ^ | ^  *right arm | ^  *technical jerkiness | ^  ^ |  |
| Case 6 |  |  |  |  |  |  | CP |
| V1  V2 | ^  ^ | ^  ^ | ^  ^ | *  ^ | ^  ^ | ^  ^ |  |

Abbreviations: CA=corrected age, CP=cerebral palsy, n=number of participants, NDO=neurodevelopmental outcome, TD= typically developing, V1=video 1 (12.0=13.6 weeks), V2=video 2 (14.0-15.6 weeks), *=reason for exclusion, ^=passed
