# Supplementary material for: Reproducibility of the Motor Optimality Score–Revised in infants with an increased risk of adverse neurodevelopmental outcomes
Source: Dev Med Child Neurol. 2025 Feb 10;67(9):1176–85. doi: 10.1111/dmcn.16256 (PMC12336395; doi:10.1111/dmcn.16256)
Supplement: Supplementary file 3 — Table S2: Total MOS‐R and subcategory scores by neurodevelopmental outcome at 2 years. [file DMCN-67-1176-s003.docx]

Table S2: Inter-assessor agreement for MOS-R sub-category ‘observed postural patterns’, agreement (%) and 95% CIs, n=30 infants

|  | Postural Patterns | | | | | |
| --- | --- | --- | --- | --- | --- | --- |
|  | Head  Centered  % (95%CI) | Body  Symmetry  % (95%CI) | Asymmetric Tonic Neck posture  % (95%CI) | Variability  of Fingers  % (95%CI) | Number of typical patterns  % (95%CI) | Number of atypical patterns  % (95%CI) |
| A-B-C-D-E-F | 92.2 (87.4-95.3) | 84.7 (75.3-86.7) | 88.3 (82.8-92.2) | 84.7 (75.3-86.7) | 63.3 (56.1-70.0) | 48.3 (41.1-55.6) |
| Gr 1 (A-B-C) | 92.2 (84.8-96.1) | 84.4 (75.6-90.5) | 92.2 (84.8-96.1) | 85.5 (76.8-91.4) | 62.2 (51.9-71.5) | 36.7 (27.4-47.0) |
| Gr 2 (D-E-F) | 94.4 (87.6-97.6) | 85.6 (76.8-91.4) | 87.8 (79.4-93.0) | 83.3 (74.3-89.6) | 57.8 (47.4-67.5) | 46.7 (36.7-56.9) |
| Consensus* | 92.4 (86.7-95.8) | 84.1 (76.9-89.4) | 90.1 (84.8-94.7) | 83.3 (76.1-88.7) | 66.7 (58.3-74.1) | 53.3 (44.6-61.3) |
| Age at assessment |  |  |  |  |  |  |
| T1(12.0-13.6w) | 92.3(86.9-95.9) | 81.5 (74.1-87.1) | 88.9 (82.5-93.1) | 84.4 (77.4-89.6) | 60.7 (52.3-68.6) | 51.1 (42.8-59.4) |
| T2(14.0-15.6w) | 92.3(86.9-95.9) | 88.9 (82.5-93.5) | 90.4 (84.2-94.3) | 75.6 (67.7-82.0) | 66.7 (58.4-74.1) | 45.2 (37.0-53.6) |
| Gestational age |  |  |  |  |  |  |
| VPT | 89.6 (83.3-93.7) | 82.9 (75.7-88.4) | 88.1 (81.6-92.6) | 81.5 (74.1-87.1) | 63.0 (54.6-70.6) | 54.1 (45.7-62.3) |
| non-VPT | 95.6 (90.6-97.9) | 87.4 (80.8-92.0) | 91.1 (85.1-94.8) | 77.8 (70.1-84.0) | 64.4 (56.1-72.0) | 48.9 (40.6-57.2) |
| Outcome at 2 years |  |  |  |  |  |  |
| Typically developing | 96.7 (90.7-98.9) | 83.3 (74.3-89.6) | 93.3 (86.2-96.9) | 76.7 (66.9-84.2) | 63.3 (53.0-72.5) | 52.2 (42.0-62.2) |
| ad-NDO | 90.0 (82.0-94.6) | 90.0 (82.0-94.6) | 93.3 (86.2-96.9) | 75.5 (65.7-83.4) | 67.8 (57.6-76.5) | 41.1 (31.5-51.4) |
| Cerebral Palsy | 91.1 (83.4-95.4) | 84.4 (75.6-90.5) | 82.2 (73.1-88.8) | 86.7 (78.1-92.2) | 60.0 (49.7-69.5) | 51.1 (40.9-61.2) |

Abbreviations: ad-NDO=adverse neurodevelopmental outcome, Gr 1=assessors scored one video, Gr 2= assessors scored two videos, MOS-R=motor optimality score-revised, n=number of participants, non-VPT=not born very preterm (>32 weeks), T1=timepoint 1, T2=timepoint 2, VPT=very preterm (<32 weeks), 95%CI=95% confidence interval, *consensus= assessor scores whose MOS-R was classified within majority agreement.
