# Supplementary material for: Reproducibility of the Motor Optimality Score–Revised in infants with an increased risk of adverse neurodevelopmental outcomes
Source: Dev Med Child Neurol. 2025 Feb 10;67(9):1176–85. doi: 10.1111/dmcn.16256 (PMC12336395; doi:10.1111/dmcn.16256)
Supplement: Supplementary file 4 — Table S3: Interassessor agreement for MOS‐R subcategory ‘observed postural patterns’, agreement, and 95% CIs. [file DMCN-67-1176-s004.docx]

Table S3: Group 1 vs Group 2 MOS-R reproducibility and time taken to score, n=30 videos, 6 assessors

|  | | | | | Developmental Outcome group | | | | |
| --- | --- | --- | --- | --- | --- | --- | --- | --- | --- |
|  | All participants  n=30 | | | Typically Developing  n=10 | | ad-NDO  n=10 | | Cerebral Palsy  n=10 | |
| Total MOS-R, mean (SD) | | |  | | | |  | | |
| Gr 1_consensus_ | 18.0 (6.6) | | | 22.6 (5.1) | | 18.9 (5.1) | | 12.5 (5.7) | |
| Gr 2_consensus_ | 18.7 (5.6) | | | 22.4 (4.1) | | 19.6 (3.5) | | 14.1 (5.6) | |
| MOS-R (+2points), % (95% CI)^**^ | |  | | |  | | | |  |
| Grp 1 | 72.2 (62.2-80.4) | | | 83.3 (66.4-92.7) | | 73.3 (55.6-85.8) | | 60.0 (42.3-75.4) | |
| Grp 2 | 86.7 (78.1-92.2) | | | 90.0 (74.4-96.5) | | 93.3 (78.7-98.2) | | 76.6 (59.1-88.2) | |
| Mean diff in MOS-R (95% LOA)  Gr 1_Gr 2 | 0.7 (-6.7-8.1) | | | -0.2 (-3.2-2.8) | | 0.7 (-6.6-8.0) | | 1.6 (-8.7-11.9) | |
| Time taken to score (mins.secs) | | |  | | | |  | | |
| Gr 1, mean (SD) | 9.55 (2.42) | | | 9.01 (2.33) | | 10.28 (2.53) | | 10.17 (2.30) | |
| Gr 2, mean (SD) | 8.05 (2.57) | | | 7.27 (2.59) | | 7.49 (1.49) | | 8.59 (3.37) | |
| Mean difference Gr 1_Gr 2, 95% CI | 1.50 (0.48-2.45) | | | 1.19 (0.48-2.33) | | 2.47 (2.01-3.33) | | 1.25 (0.05-2.45) | |

Abbreviations: ad-NDO=neurodevelopmental outcome, Gr 1=assessors scored one videos, Gr 2= assessors scored two videos, mins=minutes, MOS-R=motor optimality score-revised, n=number of participants, SD=standard deviation, secs=seconds, 95%CI=95% confidence interval, 95% LOA=95% limits of agreement, ^**^= % of MOS-R total scores assessors scored within 2 points of each other (n=90 comparisons per group).
