# Supplementary material for: Reproducibility of the Motor Optimality Score–Revised in infants with an increased risk of adverse neurodevelopmental outcomes
Source: Dev Med Child Neurol. 2025 Feb 10;67(9):1176–85. doi: 10.1111/dmcn.16256 (PMC12336395; doi:10.1111/dmcn.16256)
Supplement: Supplementary file 5 — Table S4: Group 1 vs Group 2 MOS‐R reproducibility and time taken to score. [file DMCN-67-1176-s001.docx]

Table S4: Inter-assessor reliability for MOS-R total and sub-category agreement by outcome at 2 years, n=30 infants

| Outcome at 2 years CA | Subcategories | | | | | | |
| --- | --- | --- | --- | --- | --- | --- | --- |
|  | Fidgety  movements  % (95%CI) | Movement Patterns  % (95%CI) | Age-adequate  move. repertoire  % (95%CI) | Postural  Patterns  % (95%CI) | Movement Character  % (95%CI) | MOS-R Classification  % (95%CI) | Total  MOS-R  ICC (95% CI) |
| Typically Developing  (n=10 infants) | | | | | | | |
| 12 – 13=^+6^ weeks^a^ | 91.1 (79.3-96.5) | 95.6 (85.2-98.8) | 80.0 (66.1-89.1) | 71.1 (56.6-82.3) | 93.3 (82.1-97.7) | 82.2 (68.7-90.7) | 0.49 (0.13-0.90)^*^ |
| 14 – 15^+6^ weeks^a^ | 97.8 (88.4-99.6) | 97.8 (88.4-99.6) | 80.0 (66.1-89.1) | 71.1 (56.6-82.3) | 82.2 (68-7-90.7) | 86.7 (73.8-93.7 | 0.78 (0.47-0.97)^*^ |
| Total^b^ | 94.4 (87.6-97.6) | 96.7 (90.7-98.9) | 80.0 (70.6-87.0) | 71.1 (61.0-79.5) | 87.8 (79.4-93.0) | 84.4 (75.6-90.5) | 0.90 (0.77-0.97)^a^ |
| ad-NDO  (n=10 infants) | | | | | | | |
| 12 – 13^+6^ weeks^a^ | 88.9 (76.5-95.1) | 97.8 (88.4-99.6) | 75.6 (61.3-85.8) | 68.9 (54.3-80.5) | 100.0 (92.1-100.0) | 86.6 (73.8-93.7) | 0.19 (-0.05-0.77)^*^ |
| 14 – 15^+6^ weeks^a^ | 88.9 (76.5-95.1) | 97.8 (88.4-99.6) | 93.3 (82.1-97.7) | 80.0 (66.2-89.1) | 95.6 (85.2-98.8) | 88.9 (76.5-95.1) | 0.45 (0.10-0.90)^*^ |
| Total^b^ | 88.9 (80.7-93.9) | 97.8 (92.2-99.4) | 84.4 (75.6-90.5) | 74.4 (64.6-82.3) | 97.8 (92.2-99.4) | 87.8 (79.4-93.1) | 0.68 (0.24-0.91)^a^ |
| Cerebral Palsy  (n=10 infants) | | | | | | | |
| 12 – 13^+6^ weeks^a^ | 88.9 (76.5-95.1) | 88.9 (76.5-95.1) | 73.3 (57.0-84.0) | 84.4 (71.2-92.3) | 97.8 (88.4-99.6) | 86.6 (73.8-93.7) | 0.64 (0.28-0.94)^*^ |
| 14 – 15^+6^ weeks^a^ | 71.1 (56.6-82.3) | 82.2 (68.7-90.7) | 91.1 (79.3-96.5) | 84.4 (71.2-92.3) | 97.8 (88.4-99.6) | 71.1 (56.6-82.3) | 0.01 (-0.71-0.40)^*^ |
| Total^b^ | 80.0 (70.6-87.0) | 85.6 (76.8-91.4) | 82.2 (73.1-88.8) | 84.4 (75.6-90.5) | 97.8 (92.3-99.4) | 78.9 (69.6-86.0) | 0.74 (0.37-0.93)^a^ |

Abbreviations: age adequate move. Repertoire=age adequate movement repertoire, CA=corrected age, CP=cerebral palsy, ICC=intraclass correlation coefficient, MOS-R=motor optimality score-revised, n=number of participants NDO=neurodevelopmental outcome, 95%CI=95% confidence interval, ^a^n=10 videos included, ^b^n=20 videos included, *n=5 videos included.
